# 2-Acetamido-*N*-benzyl-1,4-imino-1,2,4-tride­oxy-l-xylitol (*N*-benzyl-l-XYLNAc)

**DOI:** 10.1107/S1600536810014145

**Published:** 2010-04-24

**Authors:** Sarah. F. Jenkinson, Elizabeth. V. Crabtree, Andreas. F. G. Glawar, Terry D. Butters, George. W. J. Fleet, David. J. Watkin

**Affiliations:** a Department of Organic Chemistry, Chemistry Research Laboratory, University of Oxford, Mansfield Road, Oxford OX1 3TA, England; b Oxford Glycobiology Institute, University of Oxford, South Parks Road, Oxford OX1 3QU, England; c Department of Chemical Crystallography, Chemistry Research Laboratory, University of Oxford, Mansfield Road, Oxford OX1 3TA, England

## Abstract

X-ray crystallography defines the relative configuration at the three-stereogenic centres in the title compound *N*-benzyl-l-XYLNAc, C_14_H_20_N_2_O_3_. The five-membered pyrrolidine ring adopts an envelope conformation with the N atom lying out of the plane of the other four atoms. In the crystal structure, inter­molecular O—H⋯O, N—H⋯O and O—H⋯N hydrogen bonds link the mol­ecules into chains along [100]. The carbonyl group O atom acts as an acceptor for a bifurcated hydrogen bond. The absolute configuration is determined by the use of l-glucuronolactone as the starting material for the synthesis.

## Related literature

For imino­sugars see: Asano *et al.* (2000 ▶); Watson *et al.* (2001 ▶). For the inhibition of hexosaminidases, see: Liu, Numa *et al.* (2004 ▶); Reese *et al.* (2007 ▶); Liu, Iqbal *et al.* (2004 ▶); Woynarowska *et al.* (1992 ▶). For piperidine hexosaminidase inhibitors, see: Tatsuta *et al.* (1997 ▶); Fleet *et al.* (1986 ▶, 1987 ▶); Steiner *et al.* (2009 ▶); Ho *et al.* (2010 ▶); For furan­ose hexosaminidase inhibitors, see: Usuki *et al.* (2009 ▶); Rountree *et al.* (2007 ▶, 2009 ▶); Boomkamp *et al.* (2010 ▶). For strategies for cancer treatment, see: Kato *et al.* (2010 ▶); Greco *et al.* (2009 ▶). For the use of glucuronolactone as a starting material for the synthesis of imino­sugars, see: Best, Wang *et al.* (2010 ▶); Best, Chairatana *et al.* (2010 ▶).
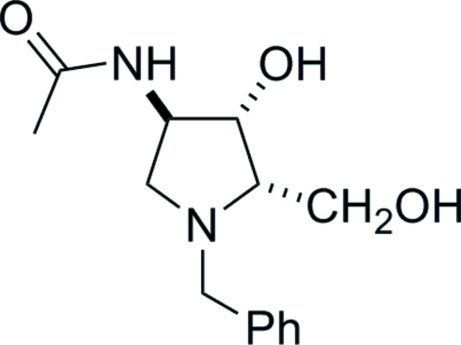


## Experimental

### 

#### Crystal data

C_14_H_20_N_2_O_3_
*M* *_r_* = 264.32
Orthorhombic, 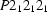

*a* = 4.9731 (1) Å
*b* = 10.0145 (3) Å
*c* = 26.9297 (7) Å
*V* = 1341.18 (6) Å^3^
*Z* = 4
Mo *K*α radiation
μ = 0.09 mm^−1^
*T* = 150 K
0.50 × 0.15 × 0.05 mm

#### Data collection

Nonius KappaCCD area-detector diffractometer
Absorption correction: multi-scan (*DENZO*/*SCALEPACK*; Otwinowski & Minor, 1997 ▶) *T*_min_ = 0.77, *T*_max_ = 1.00
7494 measured reflections
1788 independent reflections
1471 reflections with *I* > 2σ(*I*)
*R*_int_ = 0.040

#### Refinement

*R*[*F* ^2^ > 2σ(*F* ^2^)] = 0.051
*wR*(*F* ^2^) = 0.130
*S* = 0.95
1788 reflections
172 parameters
H-atom parameters constrained
Δρ_max_ = 0.33 e Å^−3^
Δρ_min_ = −0.46 e Å^−3^

### 

Data collection: *COLLECT* (Nonius, 2001 ▶); cell refinement: *DENZO*/*SCALEPACK* (Otwinowski & Minor, 1997 ▶); data reduction: *DENZO*/*SCALEPACK* and Görbitz (1999 ▶); program(s) used to solve structure: *SIR92* (Altomare *et al.*, 1994 ▶); program(s) used to refine structure: *CRYSTALS* (Betteridge *et al.*, 2003 ▶); molecular graphics: *CAMERON* (Watkin *et al.*, 1996 ▶); software used to prepare material for publication: *CRYSTALS*.

## Supplementary Material

Crystal structure: contains datablocks global, I. DOI: 10.1107/S1600536810014145/lh5029sup1.cif

Structure factors: contains datablocks I. DOI: 10.1107/S1600536810014145/lh5029Isup2.hkl

Additional supplementary materials:  crystallographic information; 3D view; checkCIF report

## Figures and Tables

**Table 1:** Hydrogen-bond geometry (Å, °)

*D*—H⋯*A*	*D*—H	H⋯*A*	*D*⋯*A*	*D*—H⋯*A*
O15—H151⋯O19^i^	0.85	1.94	2.790 (4)	173
N16—H161⋯O19^ii^	0.89	2.19	3.041 (4)	159
O1—H11⋯N4^ii^	0.85	2.29	3.121 (4)	167

Symmetry codes: (i) 
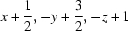
; (ii) 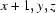.

**Fig. 1.**
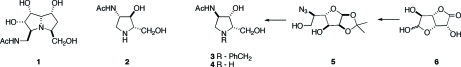
Synthetic Scheme

**Fig. 2.**
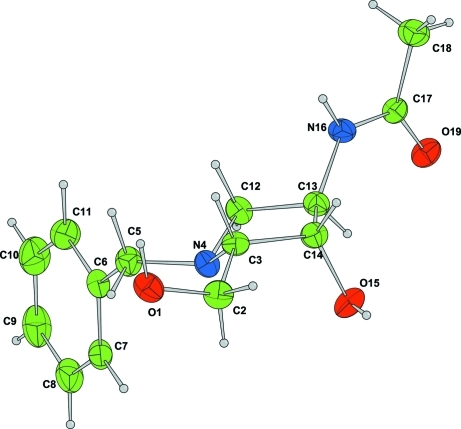
The title compound with displacement ellipsoids drawn at the 50% probability level. H atoms are shown as spheres of arbitrary radius.

**Fig. 3.**
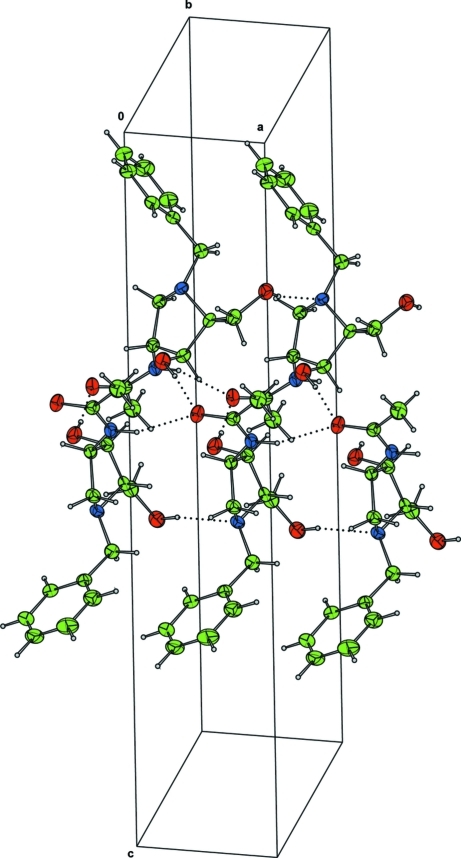
Packing diagram of the title compound with hydrogen bonds shown by dotted lines.

## supplementary crystallographic information

### Comment

Iminosugars in which the oxygen of a sugar ring is replaced by nitrogen comprise a large family of inhibitors of carbohydrate processing enzymes (Asano *et al.*, 2000; Watson *et al.*, 2001). Specific inhibition of individual hexosaminidases may allow the investigation of a number of diseases including osteoarthritis (Liu, Numa *et al.*, 2004), allergy (Reese *et al.*, 2007), Alzheimer's disease (Liu, Iqbal *et al.*, 2004), and cancer (Woynarowska *et al.*, 1992). Inhibition of *N*-acetylgalactosaminyltransferases (Kato *et al.*, 2010) and protection of macrophage activating factor (Greco *et al.*, 2009) may provide new strategies for the treatment of cancer. There are many piperidine hexosaminidase inhibitors, such as naturally occurring nagstatin (Tatsuta *et al.*, 1997) and DNJNAc (Fleet *et al.*, 1986; Fleet *et al.*, 1987; Steiner *et al.*, 2009), some with picomolar inhibition (Ho *et al.*, 2010). Until very recently, potent furanose analogue inhibitors of hexosaminidases have been unknown. The first pyrrolizidine β-hexosaminidase inhibitor, pochonicine 1 (Fig. 1) [or its enantiomer], has been isolated from a fungal strain (Usuki *et al.*, 2009). A rare example of a pyrrolidine potent hexosaminidase inhibitor is the iminoarabinitol LABNAc 2 (Rountree *et al.*, 2007; Rountree *et al.*, 2009) which has promise for the study of lysosomal storage of oligosaccharide and glycosphingolipid in iminosugar treated cells (Boomkamp *et al.*, 2010).

In a study of the hexosaminidase inhibition of diastereomers of LABNAc 2 (Fig. 1), the L-xylo-epimer L-XYLNAc 4 has been prepared from L-glucuronolactone 6, a common constituent of the chiral pool for the preparation of imino sugars (Best, Wang *et al.*, 2010). The lactone 6 may be efficiently converted to the diol 5 (Best, Chairatana *et al.*, 2010) which has been further transformed to 4*via* the *N*-benzyl L-XYLNAc 3 of L-XYLNAc. This paper reports the crystal structure of 3 which establishes the relative configuration and will allow modelling studies to rationalize enzyme inhibition by the diastereomeric 2-acetamido-pyrrolidine sugar mimics; the absolute configuration is determined by the use of L-glucuronolactone 6 as the starting material.

The pyrrolidine ring of the title compound adopts an envelope conformation with the nitrogen lying out of the plane (Fig. 2). The compound exists as chains of hydrogen-bonded molecules lying parallel to the *a*-axis (Fig. 3). Each molecule is a donor and acceptor for 3 hydrogen bonds and the hydrogen bond involving O19 is bifurcated. Only classical hydrogen bonding is considered.

### Experimental

*N*-Benzyl-L-XYLNAc 3 was crystallized from acetonitrile: m.p. 396-399 K; [α]_D_^25^ +39.9 (*c*, 0.99 in MeOH).

### Refinement

In the absence of significant anomalous scattering, Friedel pairs were merged and the absolute configuration was assigned from the use of L-glucuronolactone as the starting material.

The relatively large ratio of minimum to maximum corrections applied in the multiscan process (1:1.29) reflect changes in the illuminated volume of the crystal. Changes in illuminated volume were kept to a minimum, and were taken into account (Görbitz, 1999) by the multi-scan inter-frame scaling (*DENZO*/*SCALEPACK*, Otwinowski & Minor, 1997).

The H atoms were all located in a difference map, but those attached to carbon atoms were repositioned geometrically. The H atoms were initially refined with soft restraints on the bond lengths and angles to regularize their geometry (C—H in the range 0.93–0.98, N—H in the range 0.86–0.89 N—H to 0.86 O—H = 0.82 Å) and *U*_iso_(H) (in the range 1.2–1.5 times *U*_eq_ of the parent atom), after which the positions were refined with riding constraints.

### Crystal data

**Table:**

C_14_H_20_N_2_O_3_	*F*(000) = 568
*M**_r_* = 264.32	*D*_x_ = 1.309 Mg m^−^^3^
Orthorhombic, *P*2_1_2_1_2_1_	Mo *K*α radiation, λ = 0.71073 Å
Hall symbol: P 2ac 2ab	Cell parameters from 1650 reflections
*a* = 4.9731 (1) Å	θ = 5–27°
*b* = 10.0145 (3) Å	µ = 0.09 mm^−^^1^
*c* = 26.9297 (7) Å	*T* = 150 K
*V* = 1341.18 (6) Å^3^	Needle, colourless
*Z* = 4	0.50 × 0.15 × 0.05 mm

### Data collection

**Table:**

Nonius KappaCCD area-detector diffractometer	1471 reflections with *I* > 2σ(*I*)
graphite	*R*_int_ = 0.040
ω scans	θ_max_ = 27.5°, θ_min_ = 5.1°
Absorption correction: multi-scan (*DENZO*/*SCALEPACK*; Otwinowski & Minor, 1997)	*h* = −6→6
*T*_min_ = 0.77, *T*_max_ = 1.00	*k* = −12→12
7494 measured reflections	*l* = −34→34
1788 independent reflections	

### Refinement

**Table:**

Refinement on *F*^2^	Primary atom site location: structure-invariant direct methods
Least-squares matrix: full	Hydrogen site location: inferred from neighbouring sites
*R*[*F*^2^ > 2σ(*F*^2^)] = 0.051	H-atom parameters constrained
*wR*(*F*^2^) = 0.130	Method = Modified Sheldrick *w* = 1/[σ^2^(*F*^2^) + (0.07*P*)^2^ + 0.9*P*], where *P* = [max(*F*_o_^2^,0) + 2*F*_c_^2^]/3
*S* = 0.95	(Δ/σ)_max_ = 0.0003
1788 reflections	Δρ_max_ = 0.33 e Å^−^^3^
172 parameters	Δρ_min_ = −0.46 e Å^−^^3^
0 restraints	

### Fractional atomic coordinates and isotropic or equivalent isotropic displacement parameters (Å^2^)

**Table:**

	*x*	*y*	*z*	*U*_iso_*/*U*_eq_	
O1	0.9783 (4)	0.43009 (19)	0.61171 (7)	0.0319	
C2	0.7716 (7)	0.4811 (3)	0.58063 (10)	0.0297	
C3	0.6606 (6)	0.6164 (3)	0.59663 (10)	0.0231	
N4	0.4652 (5)	0.6138 (2)	0.63782 (8)	0.0240	
C5	0.5868 (6)	0.5705 (3)	0.68516 (10)	0.0294	
C6	0.3890 (6)	0.5636 (3)	0.72762 (9)	0.0260	
C7	0.1951 (6)	0.4640 (3)	0.72916 (10)	0.0290	
C8	0.0221 (7)	0.4529 (3)	0.76921 (11)	0.0382	
C9	0.0372 (7)	0.5432 (4)	0.80805 (11)	0.0431	
C10	0.2268 (8)	0.6428 (4)	0.80709 (11)	0.0441	
C11	0.4029 (7)	0.6540 (3)	0.76700 (11)	0.0359	
C12	0.3731 (6)	0.7532 (3)	0.64027 (10)	0.0262	
C13	0.3392 (6)	0.7942 (3)	0.58540 (9)	0.0240	
C14	0.5058 (6)	0.6899 (3)	0.55652 (9)	0.0259	
O15	0.3165 (4)	0.6041 (2)	0.53220 (7)	0.0325	
N16	0.4213 (5)	0.9324 (2)	0.57677 (8)	0.0258	
C17	0.2483 (6)	1.0276 (3)	0.56297 (10)	0.0243	
C18	0.3628 (7)	1.1653 (3)	0.55702 (12)	0.0344	
O19	0.0046 (4)	1.0055 (2)	0.55648 (7)	0.0301	
H22	0.8439	0.4905	0.5468	0.0376*	
H21	0.6258	0.4171	0.5801	0.0378*	
H31	0.8146	0.6719	0.6070	0.0290*	
H51	0.6619	0.4808	0.6798	0.0390*	
H52	0.7323	0.6330	0.6949	0.0386*	
H71	0.1814	0.4027	0.7021	0.0362*	
H81	−0.1097	0.3838	0.7705	0.0516*	
H91	−0.0852	0.5371	0.8355	0.0554*	
H101	0.2377	0.7039	0.8338	0.0523*	
H111	0.5375	0.7242	0.7665	0.0449*	
H122	0.2057	0.7596	0.6587	0.0343*	
H121	0.5034	0.8116	0.6565	0.0343*	
H131	0.1474	0.7860	0.5763	0.0293*	
H141	0.6349	0.7323	0.5324	0.0339*	
H181	0.2239	1.2282	0.5501	0.0525*	
H183	0.4944	1.1658	0.5306	0.0528*	
H182	0.4537	1.1924	0.5865	0.0527*	
H151	0.3832	0.5671	0.5065	0.0524*	
H161	0.5958	0.9521	0.5796	0.0324*	
H11	1.0957	0.4903	0.6166	0.0526*	

### Atomic displacement parameters (Å^2^)

**Table:**

	*U*^11^	*U*^22^	*U*^33^	*U*^12^	*U*^13^	*U*^23^
O1	0.0291 (11)	0.0288 (11)	0.0377 (10)	0.0048 (10)	0.0042 (10)	0.0039 (9)
C2	0.0294 (15)	0.0284 (15)	0.0313 (13)	0.0024 (14)	0.0030 (13)	−0.0026 (12)
C3	0.0188 (12)	0.0250 (13)	0.0255 (12)	−0.0023 (12)	0.0046 (11)	−0.0004 (11)
N4	0.0245 (12)	0.0263 (12)	0.0212 (10)	0.0009 (11)	0.0047 (10)	0.0021 (9)
C5	0.0259 (14)	0.0339 (16)	0.0285 (13)	0.0004 (14)	−0.0001 (13)	0.0041 (12)
C6	0.0275 (13)	0.0278 (15)	0.0228 (12)	0.0025 (13)	−0.0014 (12)	0.0050 (11)
C7	0.0293 (15)	0.0328 (16)	0.0250 (12)	0.0011 (13)	−0.0034 (12)	0.0055 (12)
C8	0.0334 (16)	0.050 (2)	0.0309 (14)	−0.0051 (16)	0.0010 (14)	0.0136 (14)
C9	0.0385 (17)	0.063 (2)	0.0279 (14)	0.0076 (19)	0.0069 (14)	0.0106 (15)
C10	0.058 (2)	0.047 (2)	0.0277 (14)	0.007 (2)	0.0017 (17)	−0.0042 (14)
C11	0.0422 (18)	0.0360 (17)	0.0295 (14)	−0.0026 (15)	−0.0002 (15)	−0.0017 (13)
C12	0.0263 (14)	0.0271 (15)	0.0251 (12)	0.0021 (13)	0.0047 (12)	0.0014 (11)
C13	0.0213 (14)	0.0231 (14)	0.0275 (13)	−0.0009 (12)	0.0012 (12)	0.0019 (11)
C14	0.0265 (14)	0.0282 (14)	0.0230 (12)	−0.0031 (13)	0.0035 (13)	−0.0009 (11)
O15	0.0286 (11)	0.0400 (12)	0.0288 (9)	−0.0021 (10)	−0.0029 (9)	−0.0099 (9)
N16	0.0213 (11)	0.0238 (12)	0.0322 (12)	−0.0029 (11)	−0.0003 (10)	0.0021 (10)
C17	0.0248 (13)	0.0254 (14)	0.0228 (12)	−0.0001 (12)	−0.0009 (12)	−0.0023 (11)
C18	0.0349 (17)	0.0257 (15)	0.0427 (16)	−0.0007 (14)	−0.0017 (16)	0.0013 (13)
O19	0.0213 (9)	0.0343 (11)	0.0345 (10)	0.0027 (10)	−0.0024 (9)	−0.0056 (9)

### Geometric parameters (Å, °)

**Table:**

O1—C2	1.420 (4)	C9—H91	0.960
O1—H11	0.850	C10—C11	1.394 (5)
C2—C3	1.525 (4)	C10—H101	0.946
C2—H22	0.984	C11—H111	0.971
C2—H21	0.967	C12—C13	1.543 (4)
C3—N4	1.475 (3)	C12—H122	0.971
C3—C14	1.517 (4)	C12—H121	0.976
C3—H31	0.987	C13—C14	1.543 (4)
N4—C5	1.476 (3)	C13—N16	1.462 (3)
N4—C12	1.471 (4)	C13—H131	0.988
C5—C6	1.510 (4)	C14—O15	1.434 (3)
C5—H51	0.984	C14—H141	1.007
C5—H52	0.992	O15—H151	0.852
C6—C7	1.388 (4)	N16—C17	1.337 (4)
C6—C11	1.396 (4)	N16—H161	0.893
C7—C8	1.384 (4)	C17—C18	1.500 (4)
C7—H71	0.954	C17—O19	1.245 (4)
C8—C9	1.385 (5)	C18—H181	0.954
C8—H81	0.954	C18—H183	0.966
C9—C10	1.373 (5)	C18—H182	0.953
			
C2—O1—H11	109.5	C11—C10—H101	120.1
O1—C2—C3	114.5 (2)	C6—C11—C10	120.3 (3)
O1—C2—H22	108.4	C6—C11—H111	119.5
C3—C2—H22	108.0	C10—C11—H111	120.2
O1—C2—H21	108.3	N4—C12—C13	104.1 (2)
C3—C2—H21	108.7	N4—C12—H122	110.6
H22—C2—H21	108.9	C13—C12—H122	112.3
C2—C3—N4	115.8 (2)	N4—C12—H121	112.4
C2—C3—C14	114.5 (2)	C13—C12—H121	110.0
N4—C3—C14	102.1 (2)	H122—C12—H121	107.5
C2—C3—H31	107.5	C12—C13—C14	104.1 (2)
N4—C3—H31	107.9	C12—C13—N16	111.9 (2)
C14—C3—H31	108.8	C14—C13—N16	114.2 (2)
C3—N4—C5	112.6 (2)	C12—C13—H131	108.7
C3—N4—C12	102.8 (2)	C14—C13—H131	109.7
C5—N4—C12	111.6 (2)	N16—C13—H131	108.0
N4—C5—C6	113.6 (2)	C13—C14—C3	104.0 (2)
N4—C5—H51	107.2	C13—C14—O15	106.5 (2)
C6—C5—H51	108.5	C3—C14—O15	111.5 (2)
N4—C5—H52	110.0	C13—C14—H141	112.5
C6—C5—H52	107.7	C3—C14—H141	109.9
H51—C5—H52	109.8	O15—C14—H141	112.1
C5—C6—C7	120.5 (3)	C14—O15—H151	112.1
C5—C6—C11	120.9 (3)	C13—N16—C17	122.7 (2)
C7—C6—C11	118.5 (3)	C13—N16—H161	117.8
C6—C7—C8	120.8 (3)	C17—N16—H161	119.4
C6—C7—H71	119.3	N16—C17—C18	116.2 (3)
C8—C7—H71	119.9	N16—C17—O19	122.6 (3)
C7—C8—C9	120.2 (3)	C18—C17—O19	121.2 (3)
C7—C8—H81	120.9	C17—C18—H181	110.7
C9—C8—H81	119.0	C17—C18—H183	109.9
C8—C9—C10	119.9 (3)	H181—C18—H183	110.0
C8—C9—H91	120.4	C17—C18—H182	110.7
C10—C9—H91	119.7	H181—C18—H182	108.6
C9—C10—C11	120.3 (3)	H183—C18—H182	106.8
C9—C10—H101	119.6		

### Hydrogen-bond geometry (Å, °)

**Table:**

*D*—H···*A*	*D*—H	H···*A*	*D*···*A*	*D*—H···*A*
C5—H51···O1	0.98	2.47	3.111 (4)	123
C14—H141···O15^i^	1.01	2.56	3.514 (4)	159
O15—H151···O19^i^	0.85	1.94	2.790 (4)	173
N16—H161···O19^ii^	0.89	2.19	3.041 (4)	159
O1—H11···N4^ii^	0.85	2.29	3.121 (4)	167

Symmetry codes: (i) *x*+1/2, −*y*+3/2, −*z*+1; (ii) *x*+1, *y*, *z*.
